# Spinal Cord Transcutaneous Stimulation Priming Largely Enhances Lower Limb Performance during a Simulated Power Training Session in Young Active Males

**DOI:** 10.1249/MSS.0000000000003855

**Published:** 2025-09-23

**Authors:** SIMONE ZACCARON, LARA MARI, MATTIA D’ALLEVA, JACOPO STAFUZZA, STEFANO LAZZER, ENRICO REJC

**Affiliations:** 1 Department of Medicine, University of Udine, Udine, ITALY; 2 Department of Medicine, School of Sport Sciences, University of Udine, Udine, ITALY; 3 Department of Neurosciences, Biomedicine and Movement Sciences, University of Verona, Verona, ITALY

**Keywords:** HALF SQUAT, POWER OUTPUT, POWER TRAINING, SPINAL CORD NEUROMODULATION

## Abstract

**Purpose:**

To investigate the potential efficacy of spinal cord transcutaneous stimulation (scTS) priming to enhance lower limb neuromuscular performance during a subsequent power training session.

**Methods:**

Eleven young active males (age: 21.3 ± 1.6 yr) participated in this randomized crossover, sham-controlled study. The priming protocol consisted of the application of scTS or sham stimulation (Sham) at rest and during warm-up for approximately 25 min. Force, velocity, and power, as well as electromyography (EMG) of lower limbs generated during unilateral half squats on a Smith machine, were assessed over two separate experimental sessions (scTS or Sham) for: 1) four power training sets, each including six unilateral repetitions with 2) the last (fourth) set continuing to failure.

**Results:**

Peak and mean power generated during the four power training sets preceded by scTS priming were significantly higher (11%, *P* < 0.001 and 14%, *P* = 0.008, respectively) than those generated in the Sham session. Similar trends were also shown by velocity, force, and total impulse of force. Exploratory EMG analysis revealed that scTS priming favored an overall improved activation of the vastus lateralis during the concentric phase. Higher peak and mean power outputs (13%, *P* = 0.006, and 24%, *P* = 0.014, respectively), associated with higher EMG amplitude of vastus lateralis, were promoted by scTS priming also for the last set to failure, which resulted in a similar number of repetitions between the scTS (32 ± 17) and Sham priming (32 ± 19) sessions.

**Conclusions:**

scTS priming enhanced neuromuscular outcomes during a simulated lower limb power training session. Further studies should implement scTS priming throughout a longitudinal power training intervention and assess its potential to enhance training-induced neuromuscular adaptations.

The ability to generate high levels of lower limb power output plays a key role in different human movement contexts. For example, the ability to generate higher levels of power typically results in enhanced athletic performance (1), and it may also contribute to injury prevention (2). Also, in the elderly population, lower limb power output is a relevant predictor of functional mobility as well as overall morbidity and mortality (3). Resistance training with overloads targeted at enhancing muscle power output (i.e., power training) is widely used and can promote improvements in maximal muscular power (1,4). Such training-induced performance enhancement is determined by morphological and neural adaptations, including changes in muscle architecture, enhanced neural drive, changes in motor unit recruitment and firing frequency, improved intermuscular coordination, and a reduction in neuromuscular inhibition (1).

In the past decades, the implementation of noninvasive neuromodulation techniques has been explored with the goal of improving neuromuscular performance during maximal efforts, with mixed outcomes. For example, a number of studies assessed the acute effects of transcranial magnetic stimulation and transcranial direct current stimulation (tDCS) on maximal performance of lower limbs. Voluntary isometric contractions and jumping ability showed improvements in some studies (5,6), while other authors found no effects or even performance deterioration during jumping and cycling (7,8). Conversely, much fewer studies have been devoted to the implementation of neuromodulation techniques for improving neuromuscular performance specifically during resistance training. For instance, a 20-min anodal tDCS applied over the legs’ motor representation cortex area increased by 9% the number of repetitions performed until concentric muscular failure across 10 sets of back squats within a single simulated training session compared with sham stimulation (Sham) (9). This positive adaptation was accompanied by a decreased training session, rate of perceived exertion (RPE), whereas no effects on neuromuscular performance (i.e., peak power, velocity, and force) tested at the end of the simulated training session were detected. Another study tested the effect of anodal or sham tDCS applied over the primary motor cortex during a 3-wk eccentric isokinetic training program on lower limb neuromuscular performance, showing similar improvements in peak torque between the sham and tDCS study groups (10).

Another noninvasive neuromodulation technique that has been originally implemented to promote motor recovery in individuals with paralysis by spinal cord injury is spinal cord transcutaneous stimulation (scTS) (11). In this population, spinal cord stimulation modulates and often increases the excitability of spinal circuitry so that the residual supraspinal inputs crossing the lesion (which are not functional under normal circumstances) can become functional sources of motor control. In able-bodied healthy individuals, exploratory studies have investigated the effects of spinal cord stimulation on motor control and neuromuscular performance, reporting different outcomes. Some authors suggested positive trends for the application of spinal cord stimulation compared with Sham on countermovement jumps (12) or on learning and retention of backward locomotion (13). Similarly, transcutaneous spinal direct current stimulation applied for 10 min increased maximal voluntary motor output and improved performance in a motor task that involved ballistic plantar flexions (14). On the other hand, stimulation applied at midline over the lumbosacral spinal cord appears to impair or have no effect on postural control (15,16). Overall, these findings suggest that spinal cord stimulation can conceivably increase the excitability of neuronal elements at the spinal (and possibly cortical) level, bringing the related neural networks closer to the activation threshold, thus enhancing neuromuscular performance during certain tasks (14,17,18).

In the present study, our main goal was to assess the potential efficacy of scTS priming to enhance lower limb power output during a subsequent simulated power training session in young active males. This perspective is supported by recent observations from our group (19), which showed that scTS priming counteracted exercise-induced fatigue trends, supporting lower limb performance during maximal isometric and explosive efforts. As a secondary aim, we implemented the electromyography (EMG) assessment of a subgroup of thigh muscles to explore potential motor control mechanisms associated with the expected ergogenic effect of scTS priming.

## MATERIALS AND METHODS

### Research Participants

Eleven healthy and physically active young male individuals (mean ± standard deviation age: 21.3 ± 1.6 yr; stature: 181.4 ± 6.1 m; body mass: 78.4 ± 6.6 kg; body mass index: 23.8 ± 1.6 kg·m^−2^) were recruited at the School of Sport Sciences (University of Udine, Italy) to participate in this study. Research participants were nonprofessional athletes who self-reported to practice team sports (soccer, basketball, and rugby), individual endurance sports (running and cycling), and/or related conditioning training activities for 4.1 ± 0.8 d/wk. All participants had at least a year of experience in the practice of half squat with overloads. Subjects had no history of neurological and orthopedic injuries. The experimental protocol was conducted in accordance with the Declaration of Helsinki and was approved by the Institutional Review Boards of the University of Udine (IRB# 197/2023). Before the beginning of the study, the subjects were carefully informed about its purpose and risks, and written informed consent was obtained from all of them.

### Experimental Protocol

**FIGURE 1:**
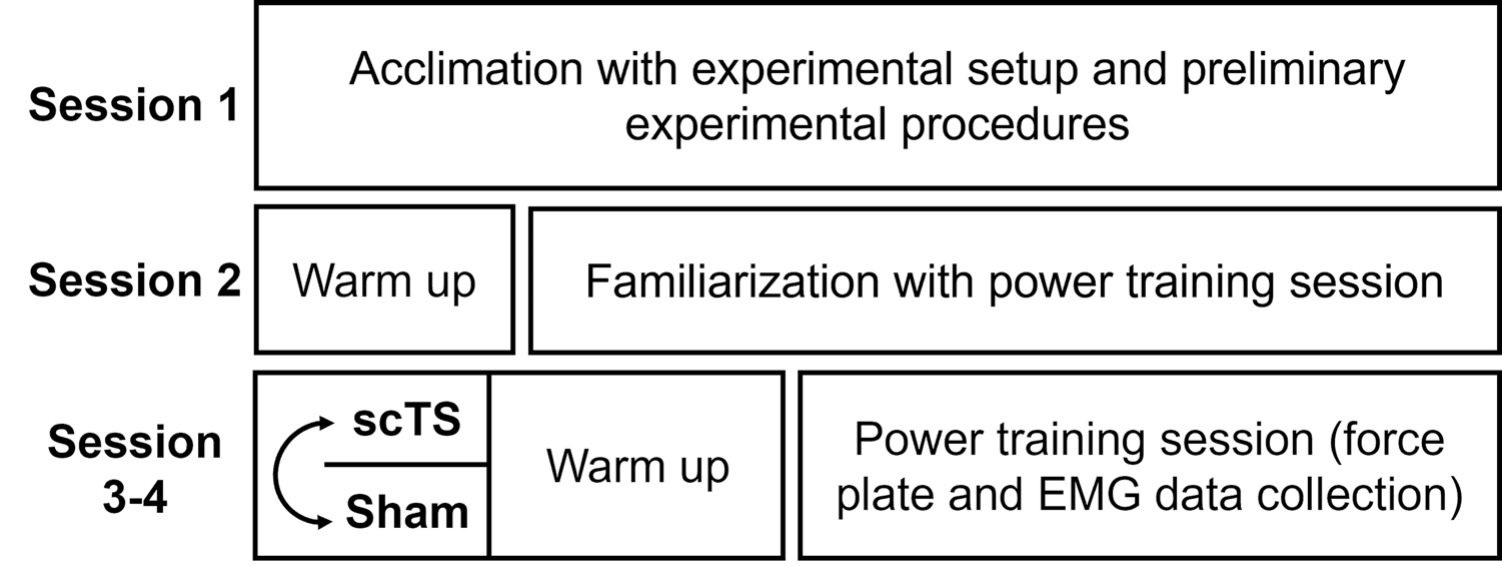
Overview of the experimental protocol. All subjects enrolled (*N* = 11) completed the experimental protocol. Testing of scTS or Sham priming in sessions 3 and 4 was proposed in a randomized order.

The experimental protocol of this randomized crossover, sham-controlled study was composed of four visits to the laboratory (Fig. 1). Experimental sessions lasted between 1 h (session 2) and 1 h 30 min (sessions 1, 3, and 4) and were performed by each participant at the same time of the day ± 1 h. The time interval between sessions 1 and 2 ranged from 2 to 4 d, and the time interval between sessions 2, 3, and 4 was 7 d. Participants were asked to wear the same gymnastic shoes at each session, to avoid any strenuous physical exercise and alcohol consumption, and to maintain their usual sleeping behavior for the 2 d before each experimental session. It was also requested that participants avoid caffeine and energy drink consumption starting 3 h before each session (9).

The first experimental session was devoted to the assessment of anthropometric characteristics, selection of the overload to be used during the unilateral half squat exercise on a Smith machine for the subsequent sessions, and the acclimation with scTS procedures and laboratory equipment. During the second experimental session, subjects practiced the entire unilateral half squat power training session. The third and fourth sessions were devoted to the assessment of kinetic and EMG parameters during the unilateral half squat power training session, which was preceded by the scTS or Sham priming. The order of scTS and Sham experimental sessions was randomized using a random number generator.

#### Anthropometric characteristics

Body mass was measured to the nearest 0.1 kg using a manual weighing scale (Seca 709, GmbH & Co. KG, Hamburg, Germany) with the subject wearing only light underwear and no shoes. Stature was measured to the nearest 0.5 cm on a standardized wall-mounted height board. The dominant lower limb was considered as the limb used to kick a ball.

#### Unilateral half squat

Subjects were asked to perform explosive unilateral half squats with overloads on a Smith machine (Multipower, Technogym, Cesena, Italy). This device is characterized by a guided barbell, which was placed across the participant’s shoulders and facilitated balance control during the unilateral explosive extensions. A rigid tactile feedback was implemented so that the participant could consistently achieve a knee angle of 100° at the end of the downward phase, which was followed by an explosive lower limb extension. The exercising limb alternated at each repetition. Therefore, immediately after the unilateral lower limb extension was completed, the foot of the uninvolved leg, which was actively flexed, was placed side by side for a bilateral stance phase lasting approximately 1–2 s. A tape was placed on the force plate to facilitate the consistent positioning of the feet throughout the half squat repetitions.

#### Selection of the overload

Participants began this part of their first experimental session with approximately 8 min of stationary cycling, joint mobilization, and half squats (six bilateral repetitions and six unilateral repetitions with 20 kg as overload) (20). The participant was then asked to perform a set of three unilateral half squat repetitions per leg. Mean propulsive upward velocity of the Smith machine barbell was recorded for each repetition by a linear position transducer (Vitruve, SPEED4LIFTS S.L., Madrid, Spain). The initial overload was 25 kg, and it was increased by 5 kg for each subsequent set (rest in between sets was 3 min) until the mean propulsive velocity for the weaker lower limb was lower than 0.45 m·s^−1^. Such overload was selected and used for the subsequent power training sessions.

#### Selection of scTS cathode site

The scTS cathode placement site was defined during experimental session 1 by means of recruitment curves assessed with the participant relaxed in supine position on a standard bedded table, as detailed elsewhere (19,21). Briefly, a constant current stimulator (DS7A, Digitimer, Hertfordshire, UK; maximal voltage: 400 V) controlled by a trigger box (GeMS TRIGGER BOX, EMS, Bologna, Italy) and related software (Direct USB for TRIGGER BOX, Version 1.00, EMS) was used to deliverer single, 1 ms monophasic square-wave pulses every 4 s. Stimulation intensity started at 5 mA and was increased up to 100 mA, or the maximum intensity that did not result in discomfort, by 5 mA increments. Five stimuli were delivered at each intensity. All participants achieved 100 mA as maximum stimulation intensity during this assessment. Two 100 × 50 mm self-adhesive electrodes (20021, Axion GmbH, Leonberg, Germany) were placed symmetrically on the skin over the iliac crests as anodes, with the longer side of the electrodes approximately aligned with the transversal plane. Also, a self-adhesive circular electrode (diameter: 25 mm; E-CM25, TensCare, Surrey, United Kingdom) used as a cathode was placed onto the skin at the thoracic (T)11-12 or T12-lumbar(L)1 intervertebral space in a randomized order, with 4 min of rest in between assessments. The stimulation site that provided the preferential recruitment of VL muscle, as defined by 1) lower stimulation intensity at motor threshold and 2) higher peak-to-peak amplitude of evoked potentials to spinal stimulation (19), was selected and implemented during the priming protocols with scTS and Sham (experimental sessions 3 and 4).

#### Priming protocol with scTS or Sham

The power training sessions considered for analysis in this study (experimental sessions 3 and 4) were preceded by the application of scTS or Sham, which were selected in a randomized order and applied during quiet standing and warm-up for a total of approximately 25 min. The constant current stimulator and electrodes used during the priming protocol are the same as those described in the section “selection of scTS cathode site.” With the participant in a standing position, the two rectangular electrodes were placed symmetrically on the skin over the iliac crests as anodes and the circular cathode electrode was placed on the skin at T11–12 (*n* = 4) or T12–L1 (*n* = 7) intervertebral space. The electrodes were secured using elastic bandages wrapped around the participant’s trunk. Additionally, a small foam piece was placed between the cathode electrode and the bandage to maintain pressure on the electrode.

During scTS priming, tonic, monophasic stimulation was delivered at 28 Hz with 1000 µs pulse width. Stimulation intensity was gradually increased to achieve the highest intensity that was well tolerated by the participant in terms of comfort and ability to perform the requested motor tasks without any restriction. On average, stimulation intensity applied during the neuromuscular priming protocols with scTS was 23.2 ± 4.2 mA, which can be also expressed as 50 ± 14% or 25 ± 5% of the stimulation intensity corresponding to VL motor threshold or highest peak-to-peak amplitude of evoked potentials, respectively, assessed during the recruitment curves in supine position (19). scTS priming was well tolerated by all participants, and it did not elicit any visible lower limb muscle contraction.

During Sham priming, stimulation pulse width was set at 200 µs, and the intensity was gradually increased for approximately 1 min, followed by a 10-s decrease and the subsequent stimulator turn off; thus, no stimulation was delivered for the remaining ~24 min of the Sham priming protocol (22). Study participants were told that two different stimulation priming protocols were tested in this study, and they were blinded to the purpose of these two different stimulations. While we did not formally assess credibility and expectance of the Sham protocol, which was developed and previously implemented by other groups, nine of the 11 participants of this study expressed surprise when we met with them individually after the completion of the study to review their outcomes and disclosed that no stimulation was applied during one of the two power training sessions.

scTS or Sham was applied during quiet standing for approximately 10 min and during warm-up for the subsequent 15 min. Warm-up activities included stepping in place, joint mobilization, unilateral balance control, and unilateral quarter squats (free movements without overload, and on the Smith machine with overload), interleaved by quiet standing. We opted to apply scTS during warm-up and standing, rather than at rest in sitting or supine position, so that spinal cord stimulation would interact with supraspinal inputs as well as weight bearing- and muscle contraction-related afferent inputs to the spinal cord (18).

#### Simulated power training session

The unilateral half squat power training session assessed in this study consisted of four sets, with 3 min of rest in between sets. At the beginning of each set, the subject was asked to place the barbell across his shoulders and stand steadily for 10 s. During the first, second, and third sets, six repetitions per leg were performed. The last (fourth) set was performed to failure. Volitional failure was defined as the inability to achieve the 100° knee flexion during the downward phase, or a mean propulsive upward velocity of the barbell higher than 0.29 m·s^−1^, for two consecutive repetitions of the same leg. Strong verbal encouragement, as well as audio and visual feedback related to barbell upward velocity, was provided at each repetition to motivate participants to exert maximal effort. During the power training sessions considered for analysis in this study (experimental sessions 3 and 4), force platform, EMG, and RPE were collected.

### Data Acquisition

Kinetic and EMG signals were collected using a dedicated acquisition system (Smart DX I, BTS Bioengineering, Milan, Italy) with a sampling rate of 1000 Hz, and the related software (Smart Motion Capture System Version: 1.10.0469, BTS Bioengineering). Ground reaction forces were collected by a force platform (Kistler, Type 9287CA, Winterthur, Switzerland).

Surface EMG was collected using a wireless EMG system (BTS FREEEMG1000, BTS Bioengineering; input impedance: 100 MΩ; common mode rejection ratio: >110 dB @50–60 Hz; sensitivity: 1 µV) and pregelled surface electrodes (BlueSensor N-00-S/25, Ambu, Penang, Malaysia) placed with an interelectrode distance equal to 20 mm. To ensure a good electrode–skin interface, before the application of the electrodes, the subject’s skin was shaved, rubbed with an abrasive paste, cleaned with an alcohol solution, and dry-cleaned with gauze. EMG electrodes were fixed at the beginning of the experimental session and were not removed until the end of the session. EMG was collected from the following lower limb muscles of the dominant lower limb: vastus lateralis (VL), at two-third on the line from the anterior spina iliaca superior to the lateral side of the patella; rectus femoris (RF), midway between the anterior spina iliaca superior and the superior part of the patella; and biceps femoris (long head, BF), midway between the ischial tuberosity and the lateral epicondyle of the tibia (23).

The RPE was assessed by means of the revised category-ratio scale (0–10 scale), which can be used to evaluate physiological and perceived stress during resistance training (24). Participants were previously familiarized with the RPE method, including its instructions and procedures. The RPE was recorded at three time points within an experimental session: 1 min before the start of the warm-up with scTS or Sham, 5 min after the end of the warm-up, and 5 min after the last set of unilateral half squat.

### Data Analysis

Data were processed using the software LabChart Reader (ADInstruments, Inc., Dunedin, New Zealand). Vertical ground reaction force signals were low-pass filtered at 25 Hz, and EMG signals were band-pass-filtered at 10–499 Hz.

#### Kinetic data analysis

The constant system mass corresponding to the combination of the subject’s body mass and the overload selected at the Smith machine was assessed at the beginning of each set during 10 s of quiet standing. The vertical ground reaction force baseline corresponding to such constant system mass was then subtracted from the vertical ground reaction force assessed during the active part of the set (i.e., net vertical ground reaction force) (25). The system mass velocity was calculated throughout the entire power training set by integrating the net vertical ground reaction force-time data using the trapezoid rule (26). To avoid the small error due to integration drift, we implemented the dedicated time constant decay function (*τ* = 1 s) to reset the system mass velocity each time the vertical ground reaction force passes through zero (27,28). Instantaneous power was obtained by multiplying the instantaneous values of net vertical ground reaction force and velocity throughout the entire sampling period (26). This procedure allowed us to investigate selected kinetic variables during the concentric phase of each unilateral half squat repetition by identifying: 1) the start of the concentric phase as the time point at which the velocity crossed zero while changing from negative to positive; 2) the end of the concentric phase as the time point corresponding to the net vertical ground reaction force decreasing to zero (26,29).

Peak and mean (concentric) force, velocity, and power generated during each repetition were considered for analysis. Since the peak velocity occurred near the end of the concentric phase time window, its value was assessed during a dedicated analysis. These kinetic variables deriving from the right and left lower limbs were finally averaged to assess the overall effects of the priming protocol with scTS or Sham. The concentric impulse of force generated during each repetition was also calculated by integrating the net vertical ground reaction force-time curve using the trapezoid rule (26), and then the total impulse of force (i.e., sum of impulses generated during all repetitions considered for analysis) was assessed.

#### EMG data analysis

EMG amplitude of VL, RF, and BF muscles was assessed during the concentric phase of each unilateral half squat repetition of the dominant lower limb by root mean square, and expressed as a percentage of the highest EMG amplitude generated within a 1-s time window during an isometric maximal voluntary contraction (MVC) of the dominant lower limb extensors at the Smith machine. During MVC, no plates (i.e., overload) were positioned onto the guided barbell. From a bipedal standing position, with the barbell placed across their shoulders, participants were asked to flex their lower limbs to achieve a knee angle of 100°. The operator positioned near the Smith machine guided this downward movement and, when the correct position was achieved, assisted the participant in rotating the barbell so that the hooks attached to it would connect to the dedicated round pins mounted on the main frame of the Smith machine, blocking any upward movement. At this stage, the participant actively flexed the nondominant lower limb and performed a maximal isometric lower limb extension with the dominant limb lasting approximately 4–5 s. Two MVC attempts were performed after a dedicated 5-min warm-up at the beginning of the experimental session (i.e., before the placement of stimulating electrodes), with 2 min of rest in between attempts.

#### Muscle recruitment data analysis

The EMG raw signal of each muscle (VL, RF, and BF) was rectified and integrated (iEMG) within each concentric phase considered for analysis. The iEMG was then expressed as a percentage of its maximal value attained at the end of each concentric phase. The percent iEMG values obtained at the four time windows corresponding to 20, 40, 60, and 80% of the concentric phase were finally considered for analysis (30).

### Statistical Analysis

Statistical analysis was performed using JASP 0.19 (University of Amsterdam, Amsterdam, The Netherlands). A *P* value less than 0.05 was considered statistically significant. Results were expressed as mean and standard deviation. The kinetic and EMG outcomes considered in this study were separately assessed for 1) the four power training sets, each including six unilateral repetitions, and 2) the entire last (fourth) set that continued to failure. The Shapiro–Wilk test was used to verify the normality of distributions. Paired *t* test or Wilcoxon test, depending on data distribution, was used to assess the effects of scTS versus Sham priming on the neuromuscular variables examined during the power training session. *P* values related to the effect of scTS versus Sham on EMG recruitment at different time points of the concentric phase were adjusted for multiple comparisons. Also, a two-way within-subjects analysis of variance was implemented to investigate the RPE scores, with “time” (i.e., before the beginning of priming protocol, after the priming protocol, and after the completion of the power training session) and “treatment” (i.e., priming protocol with scTS or Sham) as repeated measures factors. Assumption of sphericity was verified by Mauchly test. When the assumption of sphericity was not met, the significance of the *F* ratios was adjusted according to the Greenhouse–Geisser procedure. When significant differences were found, a Bonferroni *post hoc* test was used to determine the exact location of the differences. Finally, effect sizes (ES) comparing the neuromuscular outcomes obtained with the scTS or Sham priming protocol were calculated. ES values lower than 0.20 were considered negligible, between 0.20 and 0.49 small, between 0.50 and 0.79 medium, and equal to or greater than 0.80 large. The sample size for this study was estimated *a priori* using G*power (version 3.1.9.7, Henry University of Düsseldorf, Düsseldorf, Germany) (31). Based on prior findings that suggested large effects of scTS priming on lower limb power generation, we identified a sample size of 11 participants that was sufficient to detect an ES of 0.95, achieving over 80% of power for a two-sided test with matched pairs, with a significance level of 0.05.

## RESULTS

**FIGURE 2:**
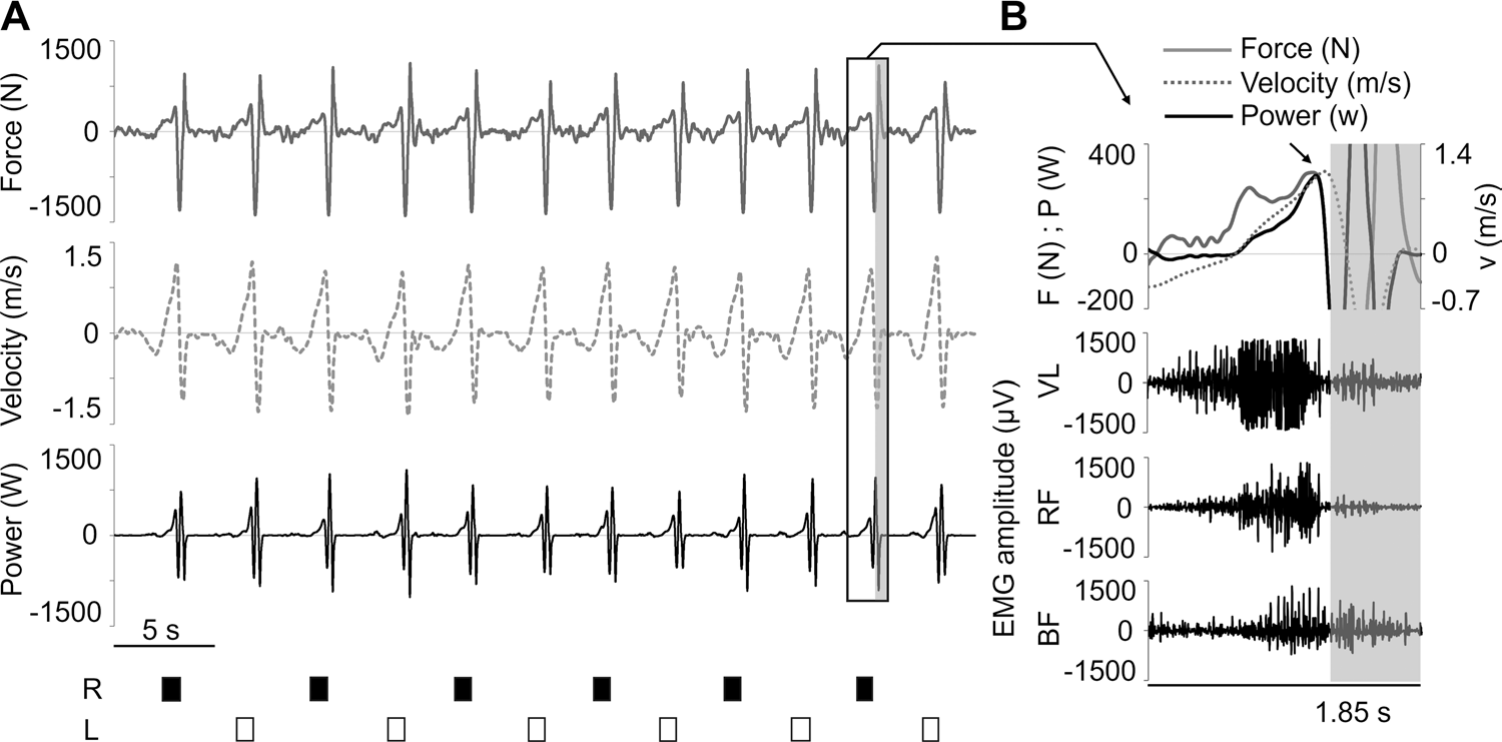
A, Time course of force, velocity, and power generated during a representative set of unilateral half squats performed at the Smith machine with an overload of 70 kg. The vertical ground reaction force baseline corresponding to the constant system mass (i.e., subject’s body mass and overload) was subtracted from the vertical ground reaction force assessed during the active part of the set. For each repetition, the exercising limb (right [R] or left [L]) is indicated at the bottom of the figure. B, Time course of force (F), velocity (v), power (P), and EMG activity of lower limb muscles during a representative repetition taken from the window depicted in panel (A). The black arrow indicates the force, velocity, and power peaks considered for analysis. The subsequent positive peaks of force and power, observed after the end of the concentric phase (gray shaded area), are related to barbell rebounds and are not considered for analysis.

Force, velocity, and power tracing throughout a set of unilateral half squats performed at the Smith machine by a representative participant with an overload of 70 kg are reported in Figure 2A, and the time course of these mechanical variables, together with EMG activity of lower limb muscles within a single half squat repetition, is exemplified in Figure 2B.

**FIGURE 3:**
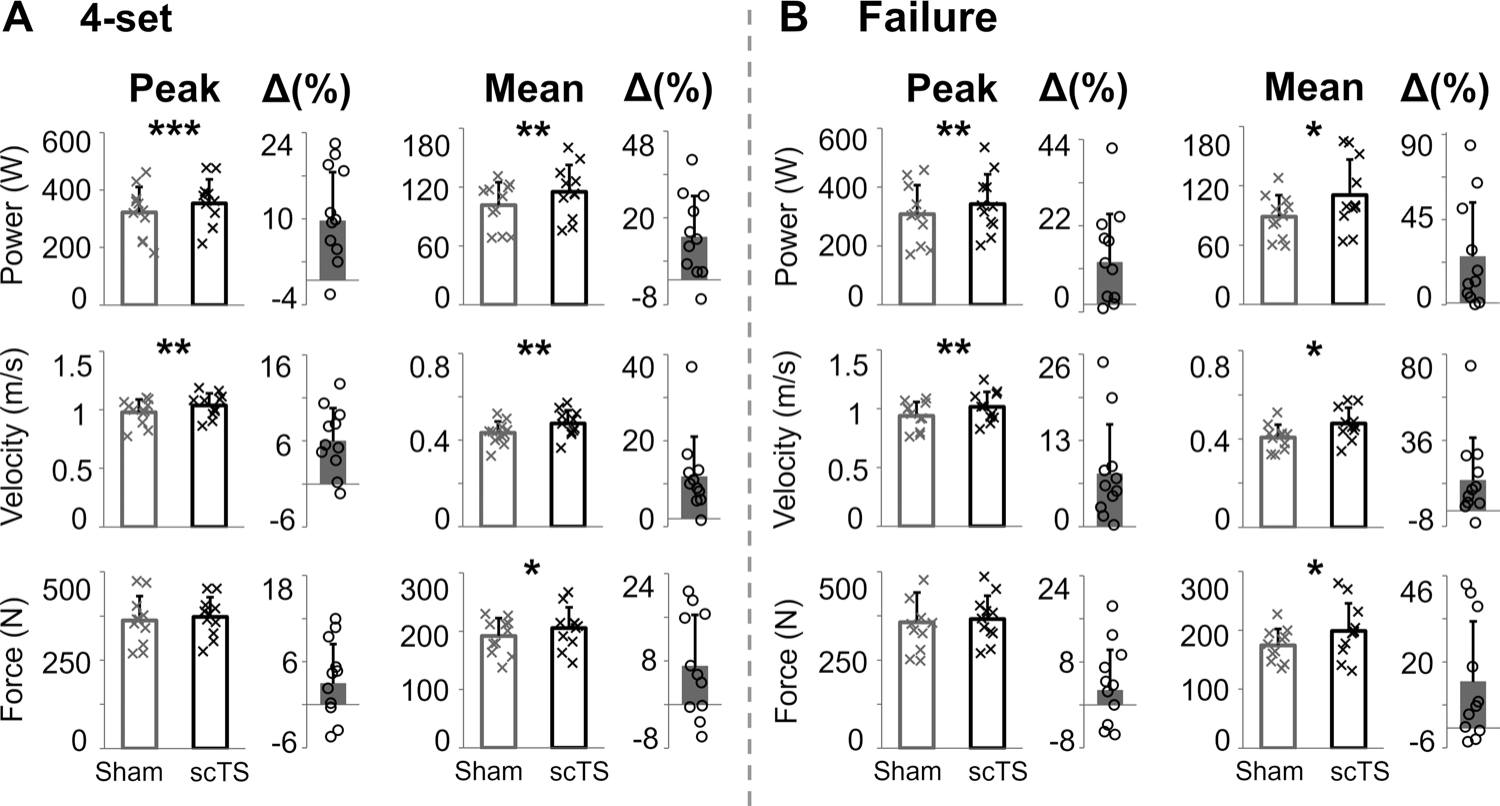
Peak and mean power, velocity, and force output generated during (A) the four unilateral half squat power training sets and (B) the entire last set to failure, following the scTS or Sham priming are reported. Results are described as individual data points (black crosses: scTS; gray crosses: Sham) as well as mean and standard deviation. The percentage difference (Δ(%)) between scTS and Sham power training sessions is also shown as individual data points (black empty circles) as well as mean and standard deviation. Differences between power training sessions preceded by scTS or Sham priming were statistically compared by paired *t* test or Wilcoxon test: *P < 0.05; **P < 0.01; ***P < 0.001.

Overall, lower limb performance during the four power training sets, each including six unilateral repetitions per leg, was largely enhanced when preceded by the scTS priming protocol (Fig. 3A). Peak and mean power output generated after the scTS priming protocol were significantly higher than those assessed in the power training session preceded by Sham priming protocol (*P* < 0.001, ES: 1.61, +11% and *P* = 0.008, ES: 0.99, +14%, respectively; Fig. 3A). Higher peak and mean velocity output (*P* = 0.001, ES: 1.35, +6% and *P* = 0.004, ES: 1.14, +11%, respectively) were also found in the power training session preceded by scTS priming protocol compared with Sham. Similar trends were observed for peak and mean force output (*P* = 0.095, ES: 0.56, +4% and *P* = 0.043, ES: 0.70, +7%, respectively). While the power training parameters (sets, repetitions, and overload) remained constant within each subject for the scTS and Sham sessions, the total impulse of force (i.e., sum of impulses generated during 48 repetitions of the power training session) was also significantly higher (*P* = 0.010, ES: 0.95, +6%) with the scTS priming protocol (6.24 ± 0.93 kNs vs 5.90 ± 0.94 kNs).

Similar positive effects of the scTS priming protocol on lower limb neuromuscular performance were also found for the last (fourth) set to failure of the power training session (Fig. 3B), which resulted in 32 ± 17 repetitions in the scTS session and 32 ± 19 repetitions during the Sham session. Specifically, we observed significantly higher peak and mean power output (*P* = 0.006; ES: 1.05; +13% and *P* = 0.014; ES: 0.90; +24%, respectively) as well as peak and mean velocity output (*P* = 0.005; ES: 1.08; +8% and *P* = 0.018; ES: 0.86; +17%, respectively) in the power training session preceded by scTS priming protocol compared with Sham. Mean force output was also higher after scTS priming (*P* = 0.029, ES: 0.77, +14%; Fig. 3B), while peak force was not significantly different between the two priming protocols (*P* = 0.230, ES: 0.39, +4%). Such enhanced neuromuscular performance with scTS priming during the last set to failure was observed with a total impulse of force that did not differ significantly between the two experimental conditions (4.15 ± 0.20 kNs vs 3.83 ± 2.27 kNs for scTS and Sham, respectively; *P* = 0.331, ES: 0.31).

**FIGURE 4:**
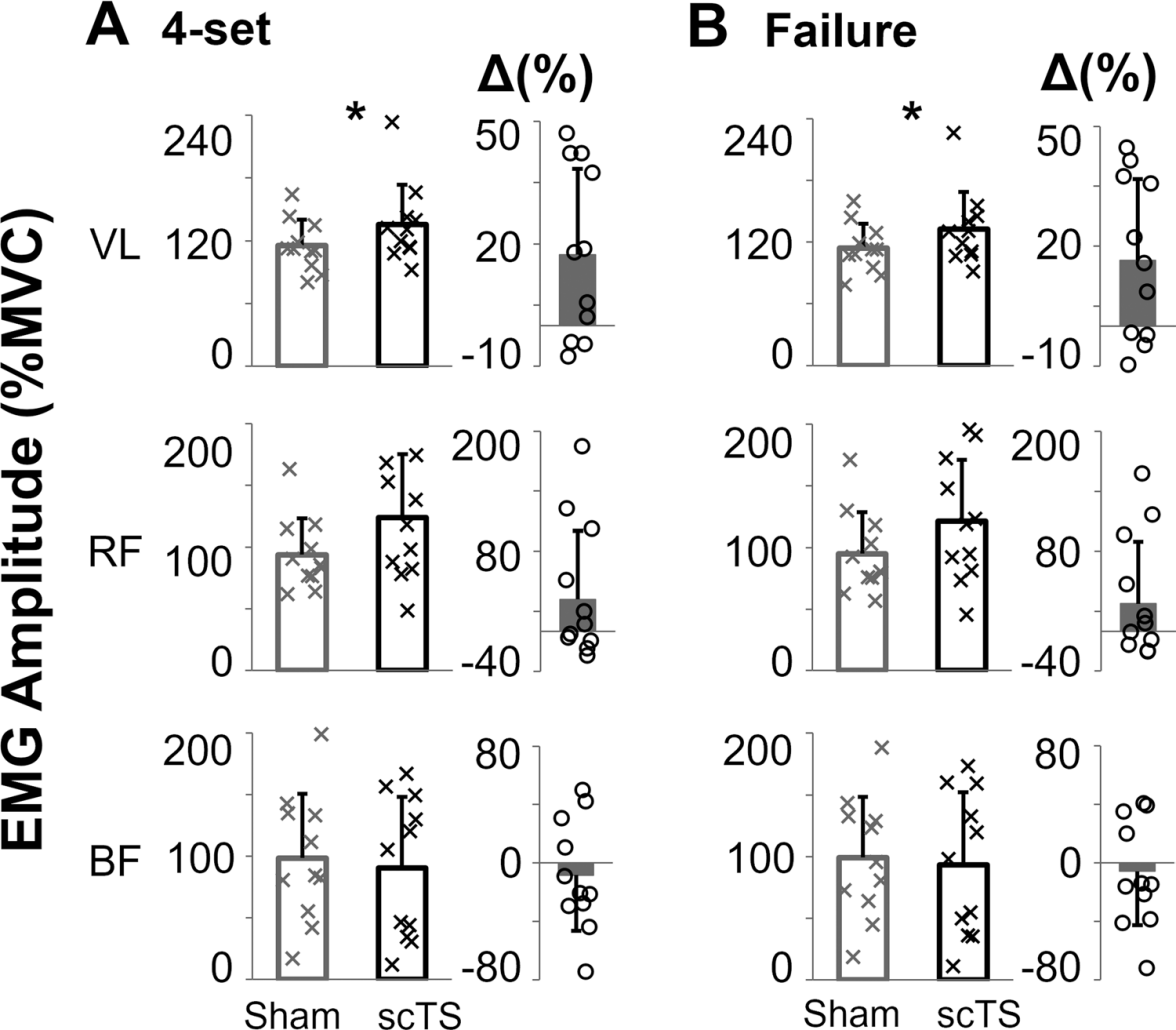
EMG amplitude of representative lower limb muscles of the dominant side generated during (A) the four unilateral half squat power training sets and (B) the entire last set to failure, following the scTS or Sham priming, is reported. EMG amplitude is quantified by root mean square and expressed as a percentage of the maximal voluntary contraction (MVC) assessed during isometric lower limb extension. Results are described as individual data points (black crosses: scTS; gray crosses: Sham) as well as mean and standard deviation. The percentage difference (Δ(%)) between scTS and Sham power training sessions is also shown as individual data points (black empty circles) as well as mean and standard deviation. Differences between power training sessions preceded by scTS or Sham priming were statistically compared by paired *t* test or Wilcoxon test: *P < 0.05.

Interestingly, the improved performance promoted by scTS priming was associated with higher EMG amplitude of VL, both during the four power training sets (*P* = 0.026, ES: 0.78, 17%; Fig. 4A) and the last set to failure (*P* = 0.029, ES: 0.77, 16%; Fig. 4B). RF showed a similar trend (*P* = 0.090 and 0.107 during the four training sets and the last set to failure, respectively), whereas no differences were noted for the BF (Fig. 4).

**FIGURE 5:**
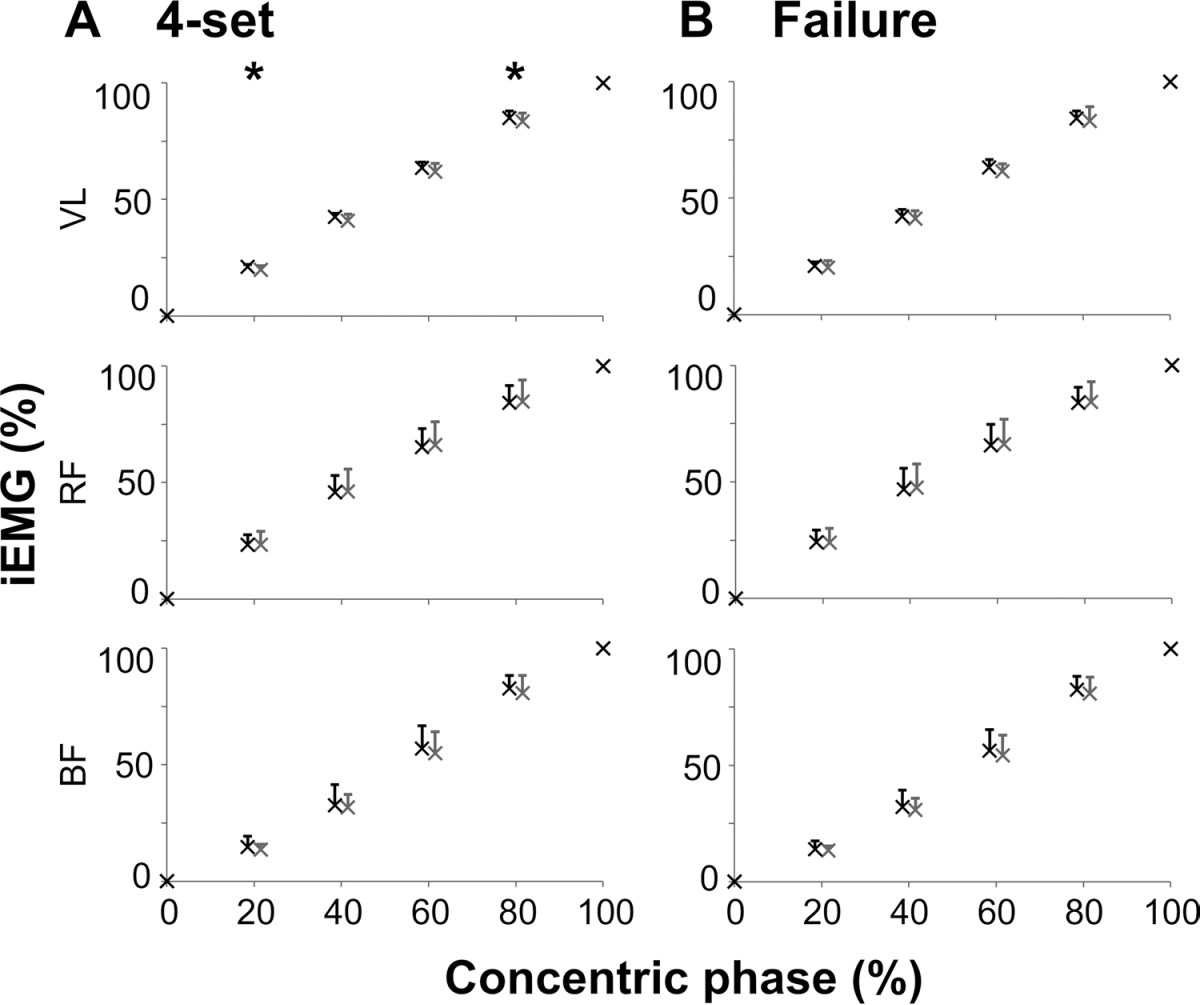
Integrated EMG (iEMG), expressed as a percentage of the maximal value reached at the end of each concentric phase of the unilateral half squats, is assessed and reported at the six indicated time points (0, 20, 40, 60, 80, and 100%) of the concentric phase. iEMG values were calculated for (A) the four unilateral half squat power training sets and (B) the entire last set to failure, following the priming protocol with scTS or Sham. Results are reported as mean and standard deviation (black crosses: scTS; gray crosses: Sham). Data points are offset by 1% along the *X*-axis to improve visualization. EMG was collected from the dominant lower limb for the following muscles: VL, RF, and BF. Differences between power training sessions preceded by scTS or Sham priming protocol were statistically assessed by paired *t* test or Wilcoxon test adjusted for multiple comparisons: *P < 0.05.

We also assessed whether the two priming protocols differentially affected the EMG recruitment of the thigh muscles subset considered for this exploratory motor control analysis (Fig. 5). It is worth noting that, during the four sets of the power training session with scTS priming, VL showed significantly higher percent iEMG at 20% (*P* = 0.028, ES: 1.02, 6.7%) and 80% (*P* = 0.040, ES: 0.84, 1.8%) of the concentric phase compared with the Sham session (Fig. 5A). No differences in muscle recruitment were noted during the last set to failure (Fig. 5B).

Finally, RPE assessed throughout the power training sessions was not affected by the type of priming (treatment *P* = 0.890; time × treatment *P* = 0.537). RPE did not change significantly before and after warm-up (scTS: 1.8 ± 1.0 and 2.0 ± 1.4; Sham: 1.5 ± 0.6 and 2.3 ± 1.0; *P* = 0.100), whereas it was significantly higher than these two time points after the completion of the power training session (scTS: 6.5 ± 2.5, and Sham: 6.3 ± 2.1; *P* < 0.001).

## DISCUSSION

scTS priming significantly enhanced lower limb neuromuscular performance during a subsequent simulated power training session consisting of unilateral half squats in young active males. Peak and mean power outputs generated during four power training sets, each including six unilateral repetitions, were significantly larger (11% and 14%, respectively) when scTS priming was applied compared with Sham. scTS priming also promoted significant enhancement in neuromuscular performance during the last (fourth) power training set to failure, with peak and mean power outputs that were 13% and 24% higher, respectively, compared with the Sham session. These findings were associated with an overall improved activation of the VL (i.e., higher EMG amplitude and recruitment) during the concentric phase.

### Enhancement of Lower Limb Performance during Power Training by scTS Priming

Lower limb power output is of paramount importance to support athletic performance in many sports, and it appears to be an even more critical determinant of physical functioning in the elderly population compared with muscle strength (2). Physical training programs with overloads are among the most common options to improve muscle power. In this study, we implemented a simulated power training focused on unilateral half squat with overload, which is generally chosen to improve some aspects of athletic performance (i.e., sprinting, jumping, and changing direction) and maximize the transfer of training adaptation to performance (32). Here, the application of scTS before the power training session significantly and largely improved peak and mean power generated (11% and 14%, respectively; Fig. 3A).

During power training, developing the ability to express high forces in very short periods, and to express high forces as the velocity of shortening increases, is the key aspect to maximize power gains (33). The intent to move the overload with high velocity is another important component of power training, as it contributes to reaching a greater potential for developing higher power across various loads (34). In the present study, strong verbal encouragement, as well as audio and visual feedback related to barbell upward velocity, was provided throughout the power training sessions to motivate participants to exert maximal efforts and achieve the highest velocity. It is worth noting that scTS priming promoted significantly higher peak and mean system mass velocity throughout the power training session (6% and 11%, respectively) compared with sham priming (Fig. 3A).

Another relevant parameter of power training is the overload selection. In the present study, the overload corresponded to 77 ± 14% bodyweight and was selected during the first experimental session (Fig. 1), taking into consideration the procedures suggested by Escobar Hincapié et al. (20). Briefly, the overload was progressively increased until the mean propulsive velocity of the weaker lower limb dropped below 0.45 m·s^−1^. A limit of the present study is that we did not directly assess the relationship between the selected overload and the one-repetition maximum (1RM). While many investigators support the idea of using an optimal overload to maximize maximum muscular power, there is inconsistency in its determination. For example, optimized power development can be generated with overloads ranging between 30% and 70% 1RM during back squat or between 45% and 70% 1RM during half squat (33,35).

The improvements in lower limb neuromuscular performance promoted by scTS priming that are mentioned earlier are related to the primary portion of the simulated power training session, which is the four sets of six repetitions per leg. Our selection of repetitions and sets is in line with other training protocols reported in the literature for similar goals (4,33,36). Furthermore, we also tested the effects of scTS priming during the last training session set to failure, finding similar significant and large enhancement in lower limb power generation (13% and 24% for peak and mean power, respectively; Fig. 3B) compared with Sham priming (Fig. 3B). Notably, such improvement in power output was achieved while the number of half squat repetitions was similar and the total impulse of force was not different (*P* = 0.331) between experimental conditions. Such a positive effect of scTS priming is also relevant because practitioners may choose to achieve failure, particularly at the end of a power training session, with the goal of recruiting a larger number of motor units, possibly leading to a more effective training stimulus (37).

### Potential Mechanisms Supporting the Ergogenic Effect of scTS Priming

Experimental and computational studies support the view that spinal cord stimulation recruits primarily large, myelinated fibers associated with somatosensory information at their entry into the spinal cord, which in turn modulates the excitability of the spinal circuitry controlling muscle activation (38,39). In the present study, the application of scTS, which occurred before the power training session and lasted approximately 25 min, conceivably increased the excitability of neuronal elements at the spinal level, bringing the related neural networks closer to the activation threshold (14,17,40,41). Previous observations in physically active individuals suggest that such neural adaptations may have contributed to the enhanced neuromuscular performance observed during the simulated power training assessed in the present study (Fig. 3). In particular, multiple within-session assessments of maximal lower limb performance showed trends of increased or maintained performance when spinal cord stimulation priming preceded the motor task, whereas trends to decrease performance occurred in the session where Sham was implemented. This was true for a series of vertical countermovement jumps assessed within 3 h since the application of spinal stimulation or Sham (12), as well as for MVC of knee extensors assessed before and after 25 min of an exercise-based protocol performed with scTS or Sham (19). Further, such opposite trends for MVC were accompanied by a significant decrease in EMG amplitude of knee extensors in the Sham session, and no change in the scTS session. These findings were interpreted as spinal cord stimulation priming conceivably promoted modulatory effects to mitigate central fatigue mechanisms, which can contribute to approximately 20%–25% of neuromuscular performance impairment (42,43).

In the present study, the EMG analysis of representative thigh muscles supports the mechanistic hypothesis reported earlier, in that scTS priming promoted the improved activation of VL, a key knee extensor, in terms of both its amplitude (Fig. 4) and recruitment (Fig. 5A), which could be interpreted as signs of increased neural excitability (44). RF also showed trends of increased EMG amplitude with scTS priming (Fig. 4). A limitation of the present study is that we did not have the capability to expand EMG analysis to additional muscles primarily involved in unilateral half squat, such as gluteus maximus, vastus medialis, and adductors. Future investigations, including the analysis of more muscles and their coordination, may provide further insight into this important topic.

In this study, we opted to apply scTS during approximately 25 min of quiet standing and warm-up, rather than at rest, so that afferent inputs (i.e., weight bearing and muscle contraction related) and supraspinal inputs to the spinal cord would be integrated with spinal cord stimulation to prime the nervous system. The RPE analysis confirmed that the proposed approach did not increase the level of perceived exertion in either experimental session (scTS or Sham). Briefly, the scTS parameters implemented were selected with the main goal of facilitating the increased excitability of the knee extensors’ motor pools together with a larger neural network including interneurons (18). Further, scTS intensity was always limited by participants’ comfort, and no visible muscle contraction was elicited. Another aspect of the proposed approach that could be improved with further studies is the optimization of scTS priming duration.

While the findings of this study derive from a group of physically active young male individuals and may not be generalized to other populations, they can still provide a rationale to further explore the proposed scTS priming approach. For example, lower limb power output is a significant predictor of functional performance in elderly individuals (45), and its age-related decline is partly due to multiple impairments within the central nervous system, resulting in the decreased ability to voluntarily activate the muscle (i.e., reduction of activation capacity) (46). Thus, it would be worth investigating whether the excitability modulation of spinal neuronal elements conceivably promoted by scTS priming might enhance voluntary muscle activation in this population. Also, our findings are consistent with recent perspectives from the spinal cord injury field in that scTS applied before activity-based training to prime the nervous system may modulate the spinal neuronal networks (47). Finally, further efforts should also be implemented to replicate the findings herein reported in larger cohorts of young individuals, increasing statistical power for data analysis.

## CONCLUSIONS

The proposed priming protocol with scTS was a feasible and effective strategy to largely enhance lower limb power output during a subsequent simulated power training session in young active males. Such improvement in neuromuscular performance is relevant because lower limb power output plays a key role in different human movement contexts ranging from athletic performance to functional mobility. From a practical standpoint, it is worth noting that scTS priming precedes the actual training session and does not interfere with its regular routine. Further efforts are warranted to implement scTS priming throughout a longitudinal power training intervention and assess its potential to augment training-induced gains in lower limb neuromuscular performance.

This study was supported by the Departmental Strategic Plan (PSD) of the University of Udine-Interdepartmental Project on Healthy Ageing (2020–2025).

The authors thank the study participants for their time and commitment; Nicola Campigotto and Paolo Di Bernardo for their contribution to data acquisition; Renzo Pozzo and Francesco Grazzina for their contribution to data interpretation. No conflicts of interest were disclosed. The datasets generated during and/or analyzed during the current study will be made available through a material transfer agreement upon reasonable request addressed to the corresponding author. The results of the study are presented clearly, honestly, and without fabrication, falsification, or inappropriate data manipulation. The results of the present study do not constitute endorsement by the American College of Sports Medicine.
